# DSCNN-LSTMs: A Lightweight and Efficient Model for Epilepsy Recognition

**DOI:** 10.3390/brainsci12121672

**Published:** 2022-12-05

**Authors:** Zhentao Huang, Yahong Ma, Rongrong Wang, Baoxi Yuan, Rui Jiang, Qin Yang, Weisu Li, Jingbo Sun

**Affiliations:** School of Electronic Information, Xijing University, Xi’an 710123, China

**Keywords:** depthwise separable convolution neural network (DSCNN), electroencephalography (EEG), epileptic seizure recognition, long short-term memory networks (LSTMs)

## Abstract

Epilepsy is the second most common disease of the nervous system. Because of its high disability rate and the long course of the disease, it is a worldwide medical problem and social public health problem. Therefore, the timely detection and treatment of epilepsy are very important. Currently, medical professionals use their own diagnostic experience to identify seizures by visual inspection of the electroencephalogram (EEG). Not only does it require a lot of time and effort, but the process is also very cumbersome. Machine learning-based methods have recently been proposed for epilepsy detection, which can help clinicians make rapid and correct diagnoses. However, these methods often require extracting the features of EEG signals before using the data. In addition, the selection of features often requires domain knowledge, and feature types also have a significant impact on the performance of the classifier. In this paper, a one-dimensional depthwise separable convolutional neural network and long short-term memory networks (1D DSCNN-LSTMs) model is proposed to identify epileptic seizures by autonomously extracting the features of raw EEG. On the UCI dataset, the performance of the proposed 1D DSCNN-LSTMs model is verified by cross-validation and time complexity comparison. Compared with other previous models, the experimental results show that the highest recognition rates of binary and quintuple classification are 99.57% and 81.30%, respectively. It can be concluded that the 1D DSCNN-LSTMs model proposed in this paper is an effective method to identify seizures based on EEG signals.

## 1. Introduction

According to the World Health Organization (WHO), epilepsy is the second most common disease of the nervous system after stroke, and there are about 50 million people affected by this disease around the world [1]. Epilepsy is a transient central nervous system dysfunction caused by the abnormal discharge of brain neurons [2]. Seizures can lead to uncontrollable parts or the whole body, loss of consciousness, and even death. However, seizures are unpredictable, which may have serious economic, physiological, and psychological impacts for patients, and bring a huge burden to their families. The *Global Epilepsy Report*, published by the WHO in 2019, points out that 25% of epilepsy can be prevented early, and 70% of epilepsy patients can be seizure-free through low-cost and effective drugs. Therefore, early detection and diagnosis are of great significance to improve the effect of epilepsy treatment and the quality of life of patients.

Electroencephalography (EEG) is a method of recording brain activity using electrophysiological indicators. It is formed by the sum of the postsynaptic potentials generated synchronously by a large number of neurons during brain activity. Electroencephalography is a commonly used non-invasive method to monitor and diagnose epilepsy. Thus, the abnormal state of the brain [3] can be effectively identified. In order to diagnose a seizure, doctors need to have a long record of the patient’s EEG signals. Electroencephalography signals usually have many different channels and artifacts, which pose some difficulties and challenges for doctors in the diagnosis of seizures [4,5]. In addition, the diagnosis result is easily influenced by the doctor’s diagnostic experience and professional level [6,7]. Therefore, the development of an automatic and timely epilepsy diagnosis system can reduce workload and improve diagnostic efficiency, which has important clinical significance [8,9].

There is much technical research based on artificial features and machine learning classifiers [10]. Generally, traditional feature extraction methods include time domain [11], frequency domain [12], and time-frequency domain [13], but these methods are time-consuming and laborious with low accuracy. Machine learning classifiers include random forest, support vector machine, and so on. In fact, feature classifier has been successfully applied to epilepsy detection tasks [14]. However, features are extracted based on limited and prior manual processing. Most importantly, the characteristics of epilepsy are different between patients and even the same patient has changes over time; therefore, it is necessary to automatically extract and learn information from EEG data. In recent years, many studies have focused on the application of deep learning in EEG signal classification, and a variety of detection methods based on EEG for automatic seizure recognition have been proposed, including the convolutional neural network (CNN) [15,16,17], recursive neural network (RNN), long short-term memory networks (LSTMs) [18,19]. Deep learning is a powerful computing tool. Relevant studies show that the accuracy, specificity, and sensitivity of the 13-layer-deep CNN algorithm in the public data of the University of Bonn reached 88.67%, 90.00% and 95.00%, respectively [16]. In order to reduce storage space and detection time, a one-dimensional pyramidal CNN model is proposed [20]. Deep unsupervised neural networks such as denoising and sparse autoencoder (DSAE) are used to automatically detect seizures in time, but important information may be missed due to the sparse strategy [21]. These algorithms based on deep learning lay the foundation for the study of seizure detection [22]. Although these methods have achieved very good test performance, how to design a robust, accurate, real-time classification model is still a great challenge. Compared with traditional machine-learning methods, deep learning can automatically extract features from EEG data [23]. In addition, stacking multi-layer deep convolutional neural networks [16] may lead to dimensional disasters, consume a lot of time and space, slow down the training speed of the model, and affect the efficiency and performance of the model. At present, the most commonly used method is the convolutional neural network, which can deeply extract target features and is widely used in the image field, so it is easier to establish and get familiar with in the research field. Meanwhile, compared with the traditional RNN, the innovation of the LSTMs lies in solving the problem of gradient disappearance, which enables the algorithm to more accurately control what important information needs to be saved in memory and what information must be deleted [24,25]. Researchers usually combine them to form the CNN-LSTMs model. However, in general, the deep learning CNN model has a large number of parameters, a large amount of calculations, and high requirements for equipment.

In this paper, a classification model based on 1D DSCNN-2LSTMs is proposed. The DSCNN is combined depthwise (DW) and pointwise (PW). Compared with conventional convolutional operation, the number of parameters and operations is relatively lower. Replacing original EEG signals with a simple network matrix as the input of DSCNN can effectively reduce the input dimension and improve training efficiency. The LSTMs usually perform better than the temporal recursive neural network and hidden Markov model (HMM). The LSTMs can be used as a complex nonlinear element to construct a larger deep neural network. The combination of DSCNN and LSTMs improves the prediction efficiency while maintaining high accuracy.

The main contributions of this paper in the detection of epileptic seizures from EEG signals are as follows:

(1) The DSCNN is used to extract spatial features from EEG signals, which can distinguish signals to the greatest extent.
(2) The output of DSCNN is regarded as the input to train the LSTMs model, which can solve the problem of gradient disappearance and gradient explosion in the long sequence training process. Moreover, the temporal characteristics of the EEG signals can be extracted by LSTMs. These features are mainly needed for modeling calculation, but also indirectly help neurologists in clinical diagnosis.
(3) The model has less pre-processing of raw data, and in the future, it may be combined with existing wearable technology and smart phones, which can accurately detect and predict the development of epilepsy seizures, providing more universal applications for patients, caregivers, clinicians, and researchers.

## 2. Materials and Methods

### 2.1. EEG Data

The public UCI epilepsy recognition dataset was used in this paper [26]. In the UCI dataset, there are five different folders, each containing one hundred files. Specifically, each file represents an EEG record sample of the subject’s brain activity. Each file is a 23.6 s record of brain activity. After visual examination of artifacts, such as muscle activity or eye movements, these segments were selected and cut out from a continuous multi-channel EEG signal. The corresponding time series were sampled as 4097 data points. Each data point is the EEG recorded value at a different time point. Now, we have 23 × 500 = 11,500 continuous EEG samples, each containing 178 data points, lasting 1 s (column), the last column representing the label Y{1,2,3,4,5}.

The EEG signals in Group A and Group B were recorded using standard cortical electrode placement protocol in five healthy volunteers who were in awake and relaxed state with their eyes open (A) and their eyes closed (B). Groups C, D, and E were obtained from intracranial EEG from epileptic patients. Group C represents the interictal EEG data from the hippocampal region. Group D represents the interictal EEG data from tumor tissues. Only group E represents the seizure activity EEG data for epileptic patients. All EEG signals were recorded using the same 128-channel amplifier system, with a standard electrode position scheme designed according to the international 10–20 system, using average common reference values. After the 12-bit analog-to-digital conversion, the data were continuously written to the disk of the data acquisition computer system at a sampling rate of 173.61 Hz. The bandpass filter was set to 0.53 to 40 Hz. The original datasets were preprocessed by the UCI, which created the data in CSV file format to simplify access to the data. This was described in detail in the literature [26].

Here are five states:

(a) First state: recording of EEG signals in healthy subjects while their eyes are open.
(b) Second state: recording of EEG signals in healthy subjects with their eyes closed.
(c) Third state: interictal EEG signals were recorded from the healthy hippocampal area of the epileptic patients.
(d) Fourth state: interictal EEG signals were recorded at the site of the epileptic’s brain tumor.
(e) Fifth state: seizure activity EEG signals were recorded from the epileptic patients.

Groups A and B are scalp electroencephalograms. Groups C, D, and E are intracranial implant electrodes. The difference between the original EEG signal waveform in the seizure state and the normal state is easy to observe, while the difference between the original EEG signal waveform in different normal states is difficult to observe.

**Figure 1:**
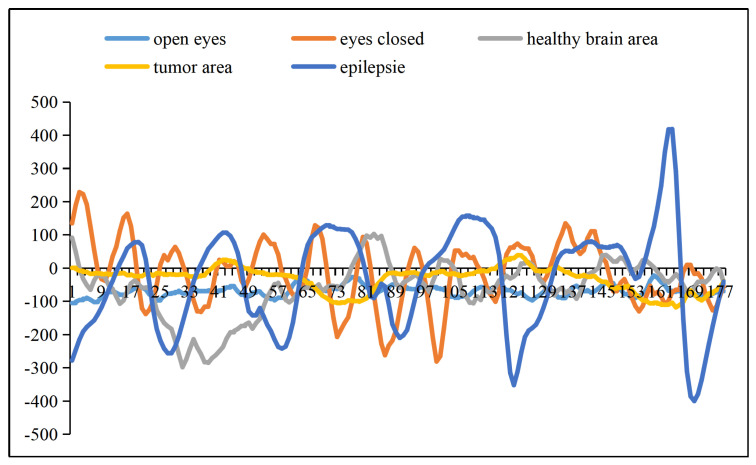
The raw electroencephalogram signal waveform of one epileptic seizure condition and four normal conditions.

Therefore, two and five groups of epilepsy recognition tasks are considered in this paper. Binaries are divided into seizures and other states, and the five classifications are all five states in the dataset. Therefore, in order to comprehensively evaluate the performance of our approach, five EEG signals are visualized and shown in Figure 1, where the X-axis is the time/s and the Y-axis is the amplitude/mV. The EEG signals from open or closed eyes and healthy brain areas have good amplitude characteristics, whereas the EEG recorded during seizures is the most periodic and high amplitude, caused by the hypersynchronous activity of a large number of neurons [26].

### 2.2. Data Pre-Processing

The UCI dataset has been pre-processed and reconstructed. Therefore, in the process of data preprocessing, it is necessary to normalize the EEG signal data, which can improve the convergence speed of the model. For normalization, the data are divided by 255. This normalization ensures the same distribution of data in the input layer. In addition, since computers cannot understand non-digital data, data labels are converted to a unique thermal code that can convert classified data into a uniform numeric format. The unique thermal coding solves the problem that the classifier is not good at processing attribute data, and also plays a role in extending the feature to a certain extent. It also facilitates the processing and computation of machine learning algorithms. After the dataset is pre-processed, the training set and the test set are divided and input into the deep learning model.

### 2.3. 1D-CNN and 1D-DSCNN

The CNN has been proven to achieve good results in decoding brain signals. As a mature neural network architecture, CNN is very suitable for automatic feature learning. It is an end-to-end learning method that can directly learn local patterns in data without any feature engineering in advance. The CNN is a feedforward neural network. This special network structure has great advantages in feature extraction and learning. The CNN has excellent performance in many applications such as image classification, target detection, and medical image analysis. The main idea of CNN is that it can take local features from higher-level inputs and transfer them to a lower level to obtain more complex features. The CNN is generally composed of convolution layer, pooling layer, and full connection layer. The convolution layer contains a certain number of convolution kernels for convolution computation of input signals. Then, the activation function is used to nonlinear the result of convolution. In the one-dimensional CNN model, rectifying linear activation unit (ReLU) is used. The pooling layer, also known as the down sampling layer, pools the output of the convolution layer to maintain a higher level of representation. Pooling process including maximum pooling and global average pooling is used in our model. After the signals pass through the convolutional layer and the pooling layer, the advanced features are fed into the fully connected layer for final classification.

The DSCNN is proposed in the literature [27]. There is a high-performance MobileNets structure in the model, and its basic principle is that the standard convolution process is divided into the depth of the equivalent convolution and point by point convolution, then through point by point convolution mixing output channel. The improved convolution model can significantly reduce the computational complexity without losing accuracy of convolution. The DSCNN can effectively decompose traditional convolution by separating spatial filtering and feature generation mechanism. The production of DSCNN is defined by two separate layers, that is, lightweight deep convolution for spatial filter and 1 × 1 point convolution for feature generation. Specifically, in depthwise separable convolution, there is only one dimension in one convolution kernel channel, and each channel is responsible for the feature graph. One channel is convolved by only one convolution kernel. After deep convolution, the number of channels in the output feature graph is the same as that in the input layer. The 1 × 1 point convolution can reduce or raise the dimension of the feature graph. The feature graph of the upper layer can be weighted and combined in the depth direction. The size of the generated new feature graph is consistent with the input data, and the main function is to combine the feature information of each channel. Since the EEG signals in this experiment are all one-dimensional features, and one-dimensional convolution filter and feature mapping are both one-dimensional, thus one-dimensional convolution is adopted in this paper by using multiple filters to carry out one-dimensional convolution.

**Figure 2:**
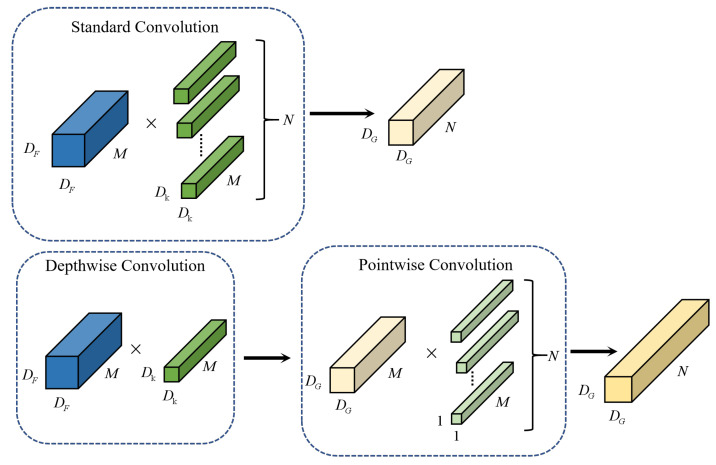
Standard Convolution process and Depthwise Separable Convolution process. D_F_, D_k_, D_G_, M and l are the dimensions, N is the number, and “×” is the multiplication formula.

For standard convolution, the dimension of input feature graph is (D_F_, D_F_, M), convolution kernel is (N, D_K_, D_K_, M), the dimension of output feature graph is (D_G_, D_G_, N), standard convolution computation quantity is (D_G_ × D_G_ × D_K_ × D_K_ × M × N). Depthwise convolution is composed of depthwise convolution and pointwise. The computation amount of depthwise convolution is (D_G_ × D_G_ × D_K_ × D_K_ × M + D_G_ × D_G_ × 1 × 1 × M × N). The process of standard convolution and depthwise separable convolution is shown in Figure 2 and Formula (1); it can be concluded that depthwise separable convolution is a much lighter convolution network.

(1) $$\frac{D_{G}\times D_{G}\times D_{K}\times D_{K}\times M+D_{G}\times D_{G}\times M\times N}{D_{G}\times D_{G}\times D_{K}\times D_{K}\times M\times N}=\frac{1}{N}+\frac{1}{D_{K}\times D_{K}}$$

### 2.4. Long Short-Term Memory Networks

**Figure 3:**
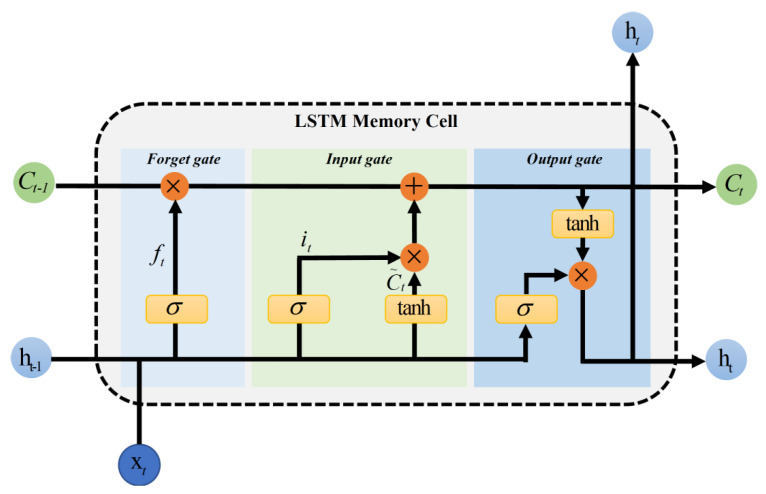
LSTMs Memory Cell. where *σ* represents sigmod activation function, tanh is an activation function, × represents multiplication, + represents addition. Other variables are temporal characteristic information.

Long short-term memory networks are a special kind of recurrent neural network (RNN). With the increase in training time and network layers, the problem of gradient explosion or gradient disappearance occurs easily in the RNN, which may lead to the inability to process long sequence data and thus an inability to obtain the information of long-distance data. Long short-term memory networks can be used in text generation, machine translation, speech recognition, generating image descriptions and video tags, and so on. As shown in Figure 3, LSTMs mainly have three gates, namely, input gate, output gate, and forget gate. At each moment, input information from the input layer will first pass through the input gate. The opening and closing of the input gate determines whether any information will be input to the memory cell at that moment. Whether information is sent out of the memory cell at any time depends on output gate. Every time a value in the memory cell is forgotten, it is controlled by forget gate. If you punch the clock, the value in the memory cell will be cleared.

The first step in LSTMs is to determine what information will be discarded from the cellular state. This decision is made through a layer called forget gate ƒ_t_. The gate reads in h_t−1_ and x_t_, usually using sigmoid as the activation function, and outputs a value between 0 and 1 for each number in the cell state C_t−1_. A reading of 1 means completely retained, 0 means completely abandoned, and most of the values of a trained LSTMs gate are very close to 0 or 1, and the rest are few. The second step is to determine what new information is stored in the cellular state. There are two parts in this step. First, the sigmoid layer, called the input gate layer, decides what value to update, and here i_t_ is regarded as the input of input gate. Then, a tanh layer creates a new candidate vector $\tilde{C}$_t_, which is obtained from input data x_t_ and hidden node h_t−1_ through a neural network layer, and is added to the state. If the previous steps have determined whether to update the old cell state, update C_t−1_ to $\tilde{C}$_t_. The updating operation is to multiply the old state with ƒ_t_, to discard the unwanted information, and to add i_t_ × $\tilde{C}$ to obtain the new candidate value. Finally, it needs to determine what the output value is, and here o_t_ represents the output gate. This output is based on cell state, but also is a filtered version. First, a sigmoid layer is run to determine which part of the cell state will be output. Next, tanh is used to obtain the cell state, a value between −1 and 1, and then multiply it by the output of the sigmoid gate. Finally, h_t_ is obtained from o_t_ of output gate and C_t_ of unit state, wherein the calculation method of o_t_ is the same as ƒ_t_ and i_t_.

The mathematical expressions of LSTMs units are defined as follows.

(2) $$f_{t}=\sigma (w_{f}\times [h_{t-1},x_{t}]+b_{f})$$

(3) $$i_{t}=\sigma (w_{i}\times [h_{t-1},x_{t}]+b_{i})$$

(4) $${\tilde{C}}_{t}=\tanh(w_{c}\times [h_{t-1},x_{t}]+b_{c})$$

(5) $$C_{t}=f_{t}\times C_{t-1}+i_{t}\times {\tilde{C}}_{t}$$

(6) $$o_{t}=\sigma (w_{o}\times [h_{t-1},x_{t}]+b_{o})$$

(7) $$h_{t}=o_{t}\times \tanh(C_{t})$$

### 2.5. 1D DSCNN-2LSTMs Model

**Figure 4:**
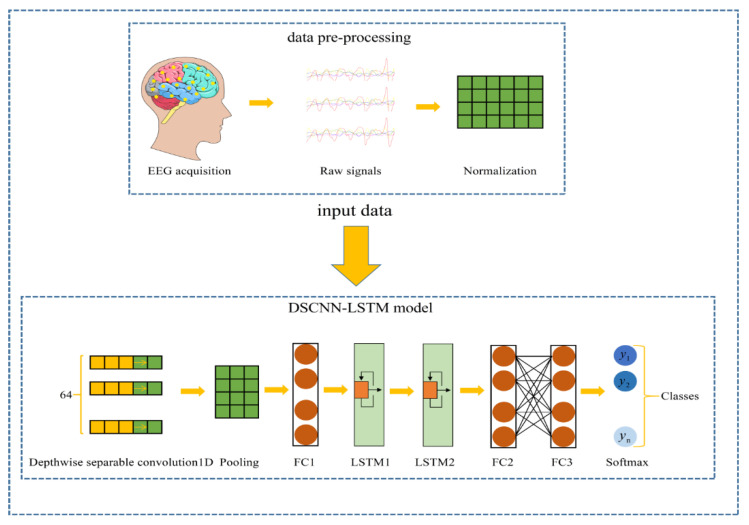
Depthwise separable convolutional neural network and two-layer long short-term memory networks (DSCNN-2LSTMs) model structure. FC is the full connection layer, and y is the classification category.

The one-dimensional DSCNN-2LSTM model proposed in this paper consists of one input layer, one depth-separable convolution layer, one pooling layer, two full connection layers, two LSTM layers, and one output layer. In order to prevent excessive fitting, drop layer is added. The detailed model structure is shown in Figure 4. It can be seen that the DSCNN-2LSTM model proposed in this paper uses very few neurons, which is the advantage of depthwise separable convolution. Table 1 shows the parameters of the DSCNN-2LSTM architecture.

Firstly, the pre-processed 1d EEG data are directly input into the input layer of the model, and the dimension of the input data is 178 × 1. Then, one-dimensional depth-separable convolution operation is performed on the input data to extract the extract features of EEG signals. The specific convolution operation is as follows: in separable Conv1D Layer1, the number of one-dimensional convolution kernels is 64, the size of convolution kernels is 3 × 1, and the step size is 1. The convolution kernels represent the sensory field of convolution. If the convolution kernels are too small and the sensory fields are insufficient, it is unable to effectively extract the association features between adjacent characters in a larger range. It is easy to ignore the association features between local adjacent characters, and the convolution kernel is too small or too large, which will adversely affect the classification results. In many tests, the appropriate convolution kernel size is 3 × 1, and the nonlinear rectification linear unit is ReLU. The ReLU activation function helps to avoid the over-fitting problem. The ReLU formula is shown in Equation (8):

(8) $$\sigma (x)={\{}_{x,x>0}^{0,x\leq 0}$$

After passing through the one-dimensional convolution layer, it enters the pooling layer, whose function is to retain the main features while reducing parameters (lowering latitude) and computation, so as to prevent over-fitting. The pooling layer then moves to the fully connected layer, where a dropout layer is added to prevent overfitting. After passing through FC Layer1, the output features are fed into the LSTMs layer, which is capable of learning useful information from EEG time series data. There are 64 neuron units in both LSTMs Layer1 and LSTMs Layer2. After the characteristics pass through the LSTMs layer, the output characteristics are sent to another FC Layer2. The FC Layer2 has 32 neurons, and finally retains the final data extracted from the whole model to the features, and then inputs the features to softmax layer for classification. Softmax classifier first converts the prediction results of the model to the exponential function, so as to ensure the non-negative probability. To make sure that the sum of the probabilities of each prediction is equal to 1, normalization needs to convert the result. The method is to divide the converted result by the sum of all the converted results, which can be understood as the percentage of the total number of converted results. That gives an approximate probability. In this way, the final feature vector will be mapped to the value of (0,1), and the cumulative sum of these values is 1, satisfying the nature of probability. When the output node is finally selected, the node with the maximum probability will be output as the target result of prediction. Softmax is shown in Equation (9).

(9) $$P(y|x)=\frac{e^{h(x,y_{i})}}{\sum_{j=1}^{n}e^{h(x,y_{i})}}$$

In this experiment, categorical_Crossentropy Loss and Adam Optimizer algorithms are used, where crossentropy is used to evaluate the difference between the probability distribution obtained by training and the real distribution. It describes the distance between the actual and the expected output probability; that is, the smaller the value of cross entropy, the closer the two probability distributions will be. Adam optimizer combines the advantages of AdaGrad and RMSProp, two optimization algorithms. The update step size is calculated by considering the First Moment Estimation and Second Moment Estimation. Adam is chosen as the optimizer because it is a simple and computationally efficient stochastic gradient descent technique [28,29]. The empirical results show that Adam is more effective than other stochastic optimization methods. The detailed configuration of the model can be adjusted according to the specific situation of the identification task. The formula of the cross entropy loss function is as in Equations (10)–(13), the derivative transformation.

(10) α=σ(z),where z=∑wj×xj+b

(11) $$C=-\frac{1}{n}\sum_{x}\left[ylna+(1-y)ln(1-\alpha )\right]$$

where *y* is the expected output; *a* is the actual output of the neuron.

(12) $$\frac{\partial c}{\partial w_{j}}=\frac{1}{n}\sum_{x}x_{j}(\sigma (z)-y)$$

(13) $$\frac{\partial c}{\partial b}=\frac{1}{n}\sum_{x}\left(\sigma (z)-y\right)$$

### 2.6. Evaluation Indicators

Now, suppose that our classification target has only two categories, which are counted as positive and negative, respectively. True positives (TP): The number of positive examples that are correctly divided; that is, the actual positive examples and the negative examples. The number of instances (number of samples) classified as positive by the classifier. False positives (FP): The number of false positives; that is, the number of instances that are actually negative but classified as positive by the classifier. False negatives (FN): The number of wrongly classified as negative examples; that is, the number of instances that are actually positive examples but classified as negative examples by the classifier. True negatives (TN): The number of correctly classified as negative examples; that is, the number of instances that are actually negative and are classified as negative by the classifier. Precision is a measure of precision and represents the proportion of examples classified as positive that are actually positive. The accuracy rate is our most common evaluation indicator. The number of pairs of samples is divided by the number of all samples. Generally speaking, the higher the accuracy, the better the performance of the model. Recall is a measure of coverage, and the measure has multiple positives that are classified as positives. F1-Score is the harmonic mean of precision and recall. The four evaluation indicators are shown in Formulas (14) to (17).

(14) $$precision=\frac{TP}{TP+FP}$$

(15) $$recall=\frac{TP}{TP+FN}$$

(16) $$accuracy=\frac{TP+TN}{TP+FP+FN+TN}$$

(17) $$f1-score=\frac{2}{\frac{1}{precision}+\frac{1}{recall}}$$

## 3. Experimental Results and Analysis

### 3.1. Experimental Setup

In this experiment, the dataset was split into 90% and 10% for training and testing, respectively. The proposed model was compared with DNN, CNN, DSCNN, LSTMs, and Bi-LSTMs and their combination models. The number of training was 100 times, and the batch size was 32. The pre-training data were all set to the same random seed and randomly shuffled and sent to the network model. Ten-fold cross-validation was also used to validate the performance of each model. The data were divided into ten parts, and take nine of them as the training set and one as the test set in turn, and the mean results of the ten times is used as the estimation of the algorithm accuracy. Both DSCNN-LSTMs and the above networks are implemented on a 12th Gen Intel (R) Core (TM) i9-12900KF 3.19 GHz processor using Python3.7.

### 3.2. The Results Analysis

#### 3.2.1. LSTMs Layer Selection

The original LSTMs model consists of a single LSTMs layer followed by an output layer. Stacking LSTMs is actually to take the output of the previous layer of the LSTMs as the input of the next layer of LSTMs, which can make the model deeper and the extracted features deeper, resulting in more accurate prediction. In order to choose the appropriate number of LSTM layers, the one-, two-, and three-layer LSTMs through 10-fold cross-validation are compared, as shown in Table 2 and Table 3. It can be seen from Table 2 and Table 3 that the average accuracy of stacking two layers of LSTMs in the two- and five-class tasks is the highest, with an average accuracy of 99.46% and 77.58%, respectively, and the accuracy begins to decline after more than two layers. Therefore, it can be concluded that DSCNN with two-layer-stacked LSTMs achieves the highest classification accuracy.

#### 3.2.2. Resolve Class Imbalances

Class imbalance refers to the situation in which the number of training examples of the different classes in a classification task varies greatly. In general, if the proportion of class imbalance is quite different, then the classifier will be greatly unable to meet the classification requirements. Therefore, before building a classification model, it is necessary to deal with the problem of classification imbalance. Clearly, the number of patients is far smaller than healthy people. There are generally solutions to solve class imbalance such as expanding the dataset, undersampling, and oversampling. Machine learning uses existing data to estimate the distribution of the entire data; therefore, more data can yield more distribution information. Undersampling is to sample the data of a large class to reduce the number of data and to make it close to the number of other classes, and then to learn. However, undersampling may lose some important information by randomly discarding large classes of samples. Oversampling is to sample the data of subclasses to increase the number of data of subclasses. However, these methods more or less affect the classification results; the skewed distribution of classes is taken into account to modify the existing training algorithm, which can be achieved by giving different weights to the majority class and the minority class. During the training process, different weight affects the classification. The overall purpose is to penalize the misclassification of the minority class by setting higher class weights while lowering the weights for the majority class. The class weight is shown in Equation (18). From the weight formula, the class weights of epilepsy and other classes can be obtained in the binary classification task, 2.5 and 0.625, respectively. The reliability of our training model is further verified by adjusting the class weights and ten-fold cross-validation. The experimental results are shown in Table 4. It can be seen from Table 2 and Table 4 that our model has high classification accuracy regardless of whether the class weight is adjusted or not, and the average accuracy exceeds 99%, which shows that our model is suitable for the EEG prediction of epilepsy and has good diagnostic performance.

(18) $$class\;weight=\frac{1}{classification\;number}\times \frac{total\;number\;of\;samples}{number\;of\;samples}$$

#### 3.2.3. Ablation Experiments

Ablation experiments have important implications for identifying accuracy and speed improvements for data augmentation, which are conducted to evaluate the performance of our algorithm on two- and five-class recognition tasks. The results of a single ablation experiment are shown in Table 5 and Table 6. It can be seen from the four evaluation indicators that the performance of the DSCNN-2LSTMs model in the binary classification task is not much different from that of the DSCNN model, but it is better than the LSTMs model. On the five-class recognition task, the performance of the DSCNN-2LSTMs model is much greater than the other two models. In order to further verify the superiority of the DSCNN-2LSTMs model for epilepsy classification results, ten-fold cross-validation is also performed. The experimental results are shown in Table 7 and Table 8. The average accuracy of our proposed model is still greater than that of the other two models. It shows that the combined model of DSCNN and LSTMs performs better than the DSCNN model and LSTMs model alone.

#### 3.2.4. Binary Recognition Task

To further verify the classification performance of the proposed DSCNN-2LSTMs for seizure detection, comparison is conducted among DSCNN-2LSTMs with other deep learning models and traditional machine learning models. The same random seed is used to ensure the trained model and the test dataset are consistent. The deep learning models include Convolutional Neural Network(CNN), Deep neural network(DNN), and bidirectional LSTMs and their combined models. Bidirectional LSTMs are an extension of traditional LSTMs, which train two models on the input sequence. The first in the input sequence is the original sample and the second is the reversed sample of the input sequence. Traditional machine learning models include AdaBoost, K Nearest Neighbors (KNN), Random Forest, and Support Vector Machine (SVM). The experiment can be seen from Table 9. When testing the validation set, the DSCNN-2LSTMs performance in this paper is the best, with an accuracy rate of 99.57%, precision of 98.79%, a recall rate of 98.79%, and an F1 score of 98.79%. The accuracy rate of Bidirectional DSCNN-LSTMs is 99.57%, second only to DSCNN-2LSTM. The DNN model has the worst performance, with 96.35% accuracy, 95.18% precision, 87.50% recall, and 91.18% F1 score evaluation. The comprehensive performance of the four traditional machine learning models is weaker than that of the deep learning model, which indicates that the deep learning model is more suitable for seizure detection than the traditional machine learning model. Among them, SVM performed the worst, with an accuracy rate of 82.26%, a precision rate of 85.55%, a recall rate of 82.23%, and an F1 score of 75.78%.

**Figure 5:**
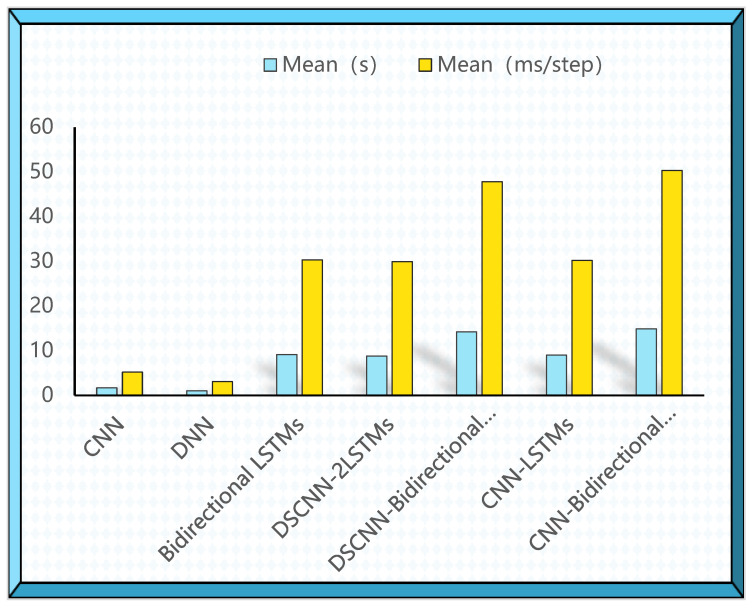
The training time of DSCNN-2LSTM and other models on the binary classification task for each iteration step size. CNN, Convolutional Neural Network; DNN, Deep neural network.

To compare the time complexity of the proposed DSCNN-2LSTMs for epilepsy detection with other models, the time complexity refers to the amount of computation required to execute the algorithm. All models are tested individually in the same environment and the iterative training average time for each model and the training average time per iteration step over ten iterations are calculated. The experimental result is shown in Figure 5. The time complexity required by DNN is the lowest, because there are only neural units in the DNN model, which reduces a lot of computation compared to other models. The time complexity of the CNN model is slightly smaller than that of the DNN. Compared with LSTMs, the computational cost of bidirectional LSTMs is greatly increased because bidirectional LSTMs need to obtain both forward and backward information. However, our proposed DSCNN-LSTMs model uses less time complexity under the premise of ensuring accuracy.

#### 3.2.5. Five-Class Recognition Task

**Figure 6:**
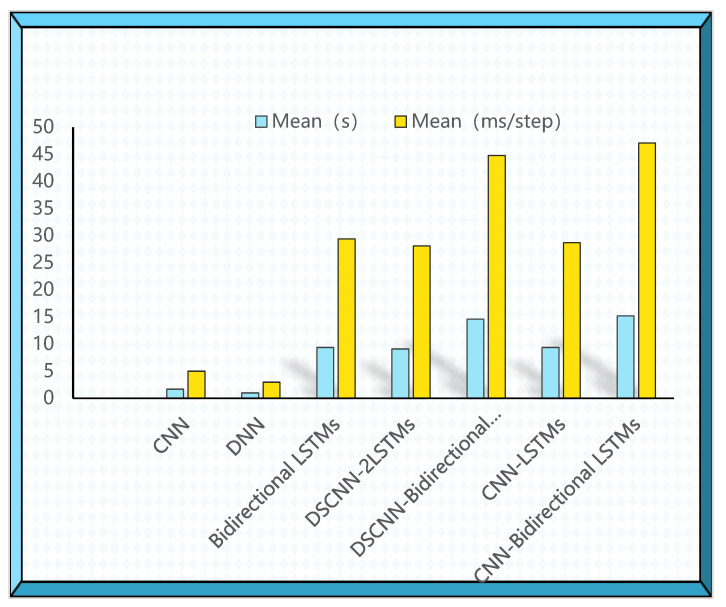
Iterative training time of DSCNN-2LSTM and other models on the five-class classification task.

Similarly, the training and testing process of applying the above model are analyzed on the quintuple classification task. The test performance of the model is shown in Table 10. It can be found from the data that the 1D DSCNN-2LSTMs model proposed in this paper has achieved the best recognition performance under different recognition tasks. The accuracy of DSCNN-2LSTMs is 81.30%, the precision is 79.21%, the recall rate is 79.95%, and the F1 score is 79.59%. The CNN-bidirectional LSTM performed worse than DSCNN-2LSTM, and SVM performed the worst in the quintubation task, with accuracy of 26.39%, precision of 33.31%, recall rate of 26.40%, and F1 score of 26.79%. We also calculate the time complexity of the deep learning model in quintuple classification tasks, as shown in Figure 6. The CNN-Bidirectional LSTMs require the highest time complexity, far higher than other models. The time complexity required by DSCNN-2LSTMs ranks in the middle among these models, but the time complexity of the combined model is the lowest. From the comprehensive evaluation indicators and time complexity, DSCNN-2LSTMs is superior to other models.

#### 3.2.6. Compare with Other Cross-Validation Models

The DSCNN-2LSTMs model proposed in this study achieves good results on binary and quintuple classification tasks. In order to further verify the accuracy advantage and stability of the model for binary classification and the quintic classification of epileptic seizures, we compared the performance of each model through the cross-validation of ten folds. The experimental results are shown in Table 11 and Table 12. In the binary recognition task, our model has an average accuracy rate of 99.46%, which is the highest among all models. The average accuracy of the DSCNN-Bidirectional LSTMs model is 98.73%, second only to DSCNN-2LSTMs. SVM performs the worst in binary recognition tasks, with an average accuracy of 81.88%. The CNN-Bidirectional LSTMs model has the highest average accuracy of 77.94% in five-class recognition tasks, 0.36% higher than our proposed model. The reason may be that CNN increases the convolution calculation compared with DSCNN. Bidirectional LSTMs capture both forward and reverse information. Compared with our model, more features are extracted, resulting in higher accuracy. However, the time complexity is much greater than our model when the accuracy is similar. SVM still performs the worst in the quintuple recognition task, with an average accuracy of 26.69%, far lower than other models. The experimental results show that our model is very effective for binary and quintuple classification tasks for epilepsy.

## 4. Conclusions

This paper presents a one-dimensional, deeply separable convolutional neural network for the detection and diagnosis of epilepsy based on EEG signals. The experimental results show that the proposed method consumes fewer computing resources, realizes the high-precision classification of seizures, and can use the original EEG data to realize real-time detection, which is helpful to the development of wearable and implantable EEG detection devices. However, the model could not predict seizures in advance. Future studies could establish multi-channel electrode DNN [30] or multi-bipolar channel input CNN [31] and a multi-classifier ensemble learning model to classify tasks under non-fixed-scale input. Pre-seizure EEG data could also be collected to train a model that could predict seizures in advance, which is crucial for epilepsy patients. Deep learning would also be applied to predict clinical drug response [32] and predict the prognosis of epilepsy surgery [33], so as to further improve the prognosis of patients and improve their living conditions.

## Figures and Tables

**Table 1:** Parameters of the DSCNN--2LSTM architecture.

Layer	Type	Output Shape	Parameters
separable_conv1d	Separable_Conv1d	(None, 176, 64)	131
max_pooling1d	MaxPooling1D	(None, 88, 64)	0
dense_1	Dense	(None, 88, 256)	16,640
dropout	Dropout	(None, 88, 256)	0
lstm_1	LSTM	(None, 88, 64)	82,176
lstm_2	LSTM	(None, 64)	33,024
dense_2	Dense	(None, 64)	4160
dense_3	Dense	(None, 6)	390

**Table 2:** The performance of DSCNN-1LSTM, DSCNN-2LSTM, and DSCNN-3LSTM model on the binary classification task.

Methods	K1	K2	K3	K4	K5	K6	K7	K8	K9	K10	Mean
DSCNN-1LSTM	97.65%	98.26%	98.00%	97.91%	98.43%	98.35%	98.17%	98.43%	98.70%	80.00%	96.39%
DSCNN-2LSTM	99.57%	99.13%	99.57%	99.65%	99.13%	99.30%	99.48%	99.39%	99.48%	99.91%	99.46%
DSCNN-3LSTM	98.78%	98.26%	98.70%	98.26%	97.91%	98.35%	98.7%	81.22%	98.87%	98.61%	96.76%

**Table 3:** The performance of DSCNN-1LSTM, DSCNN-2LSTM, and DSCNN-3LSTM model on the five-class classification task.

Methods	K1	K2	K3	K4	K5	K6	K7	K8	K9	K10	Mean
DSCNN-1LSTMs	70.52%	72.78%	72.87%	72.00%	77.22%	76.00%	74.78%	78.52%	75.48%	74.52%	74.46%
DSCNN-2LSTMs	75.91%	75.48	77.39%	77.74%	80.78%	76.43%	79.65%	74.96%	76.35%	81.13%	77.58%
DSCNN-3LSTMs	76.52%	68.61%	79.04%	73.04%	74.09%	78.09%	74.09%	78.00%	77.22%	81.30%	76.00%

**Table 4:** The Performance of DSCNN-2LSTM model after adjusting classification weight on the binary classification task.

Method	K1	K2	K3	K4	K5	K6	K7	K8	K9	K10	Mean
DSCNN-2LSTMs	99.13%	99.13%	99.48%	99.57%	99.13%	99.39%	98.17%	99.39%	99.48%	99.30%	99.21%

**Table 5:** Ablation Experiments for Binary Classification Task Recognition.

Methods	Accuracy (%)	Precision (%)	Recall (%)	F1-Score (%)
DSCNN	97.17%	96.81%	97.21%	97.01%
LSTMs	93.30%	93.00%	93.47%	93.03%
DSCNN-2LSTMs	99.57%	98.79%	98.79%	98.79%

**Table 6:** Ablation Experiments for Five-Class Classification Task Recognition.

Methods	Accuracy (%)	Precision (%)	Recall (%)	F1-Score (%)
DSCNN	74.78%	74.86%	74.65%	74.76%
LSTMs	57.57%	57.47%	57.47%	56.62%
DSCNN-2LSTMs	**81.30%**	**79.21%**	**79.95%**	**79.59%**

**Table 7:** Ablation experiment results of binary task recognition by ten-fold cross-validation.

Methods	K1	K2	K3	K4	K5	K6	K7	K8	K9	K10	Mean
DSCNN	98.09%	97.83%	98.17%	98.26%	97.74%	98.17%	97.39%	98.26%	80.78%	98.17%	96.28%
LSTMs	95.04%	97.22%	79.48%	77.83%	81.65%	97.57%	94.61%	81.22%	97.74%	96.52%	89.88%
DSCNN-2LSTMs	99.57%	99.13%	99.57%	99.65%	99.13%	99.30%	99.48%	99.39%	99.48%	99.91%	**99.46%**

**Table 8:** Ablation experiment results of five-class classification task recognition by ten-fold cross-validation.

Methods	K1	K2	K3	K4	K5	K6	K7	K8	K9	K10	Mean
DSCNN	60.70%	63.04%	64.35%	62.26%	62.35%	63.48%	64.17%	60.61%	63.74%	63.22%	62.79%
LSTM	58.09%	54.61%	55.91%	49.83%	60.26%	57.91%	58.00%	57.74%	57.13%	59.13%	56.86%
DSCNN-2LSTMs	75.91%	75.48%	77.39%	77.74%	80.78%	76.43%	79.65%	74.96%	76.35%	81.13%	77.58%

**Table 9:** The Performance of DSCNN-2LSTMs and other models on binary classification tasks.

Methods	Accuracy (%)	Precision (%)	Recall (%)	F1-Score (%)
CNN	97.13%	94.24%	92.34%	93.28%
DNN	96.35%	95.18%	87.50%	91.18%
Bidirectional LSTMs	97.96%	97.80%	97.61%	98.21%
DSCNN-2LSTMs	99.57%	98.79%	98.79%	98.79%
DSCNN-Bidirectional LSTMs	98.91%	98.60%	98.60%	98.60%
CNN-LSTMs	98.39%	98.60%	98.01%	98.40%
CNN-Bidirectional LSTMs	98.87%	98.60%	98.60%	98.60%
AdaBoost	93.60%	93.61%	93.44%	93.43%
KNN	92.21%	92.77%	92.30%	91.05%
Random Forest	97.13%	97.21%	97.42%	97.01%
SVM	82.26%	85.55%	82.23%	75.78%

CNN, Convolutional Neural Network; DNN, Deep neural network; KNN, K Nearest Neighbors; SVM, Support Vector Machine.

**Table 10:** The Performance of DSCNN-2LSTM and other models on the five-class classification task.

Methods	Accuracy (%)	Precision (%)	Recall (%)	F1-Score (%)
CNN	67.30%	67.73%	66.76%	67.05%
DNN	68.78%	67.63%	67.67%	66.91%
DSCNN	74.78%	74.86%	74.65%	74.76%
Bidirectional LSTMs	74.52%	74.75%	74.36%	74.36%
DSCNN-2LSTMs	81.30%	79.21%	79.95%	79.59%
DSCNN-Bidirectional LSTM	77.04%	77.04%	77.12%	77.08%
CNN-LSTM	78.00%	78.06%	78.06%	77.95%
CNN-Bidirectional LSTM	79.13%	79.16%	79.13%	79.36%
AdaBoost	41.73%	41.57%	42.83%	37.59%
KNN	50.65%	58. 99%	50.65%	48.34%
Random Forest	67.69%	67.52%	67.66%	67.19%
SVM	26.39%	33.31%	26.40%	26.79%

**Table 11:** The Performance of 11 models using 10-fold cross-validation on the binary classification task.

Methods	K1	K2	K3	K4	K5	K6	K7	K8	K9	K10	Mean
CNN	98.35%	97.91%	98.26%	97.74%	97.48%	98.78%	97.48%	98.35%	98.70%	98.26%	98.13%
DNN	97.22%	98.00%	97.22%	97.57%	96.70%	97.65%	96.09%	98.00%	98.26%	97.48%	97.41%
Bidirectional LSTM	98.00%	97.57%	98.26%	98.00%	97.57%	98.17%	97.57%	98.52%	99.04%	98.09%	98.07%
DSCNN-2LSTM	99.57%	99.13%	99.57%	99.65%	99.13%	99.30%	99.48%	99.39%	99.48%	99.91%	99.46%
DSCNN-Bidirectional LSTM	98.96%	98.52%	98.70%	98.78%	98.00%	99.30%	98.43%	99.13%	99.04%	98.52%	98.73%
CNN-LSTM	98.70%	98.35%	99.3%	98.96%	97.83%	98.43%	98.35%	98.87%	99.13%	98.43%	98.63%
CNN-Bidirectional LSTM	98.52%	98.43%	98.35%	99.04%	97.22%	98.61%	98.17%	98.09%	98.70%	98.52%	98.36%
AdaBoost	93.56%	94.17%	93.65%	94.78%	94.43%	94.78%	94.00%	93.91%	95.91%	94.43%	94.36%
KNN	91.91%	93.47%	92.95%	91.91%	92.17%	93.21%	92.34%	90.69%	94.78%	92.00%	92.54%
Random Forest	97.65%	97.13%	97.04%	97.65%	96.95%	97.91%	96.60%	98.26%	98.08%	97.82%	97.50%
SVM	80.60%	83.04%	81.39%	79.56%	83.21%	81.65%	81.13%	83.21%	82.69%	82.34%	81.88%

**Table 12:** The Performance of 11 models using 10-fold cross-validation on the five-class classification task.

Methods	K1	K2	K3	K4	K5	K6	K7	K8	K9	K10	Mean
CNN	72.87%	72.43%	76.09%	75.30%	74.52%	75.30%	77.22%	75.91%	74.26%	77.57%	75.14%
DNN	66.87%	66.70%	70.00%	65.83%	67.22%	69.65%	68.52%	66.78%	68.35%	66.26%	67.61%
Bidirectional LSTM	75.30%	74.09%	75.04%	74.00%	75.04%	75.48%	74.70%	73.39%	75.22%	75.13%	74.73%
DSCNN-2LSTM	75.91%	75.48%	77.39%	77.74%	80.78%	76.43%	79.65%	74.96%	76.35%	81.13%	77.58%
DSCNN-Bidirectional LSTM	77.22%	76.43%	76.52%	77.65%	71.48%	74.43%	74.61%	76.17%	74.52%	80.35%	75.93%
CNN-LSTM	73.39%	75.91%	79.22%	77.04%	78.26%	78.35%	79.13%	77.04%	78.17%	80.26%	77.67%
CNN-Bidirectional LSTM	79.04%	77.48%	81.13%	75.48%	79.65%	80.61%	78.96%	73.22%	74.70%	79.13%	77.94%
AdaBoost	43.56%	41.47%	44.17%	43.73%	42.00%	45.21%	42.78%	41.65%	44.17%	41.82%	43.05%
KNN	45.91%	50.60%	49.13%	46.43%	47.73%	48.86%	46.69%	49.04%	45.47%	46.00%	47.58%
Random Forest	70.60%	69.91%	72.95%	70.26%	70.78%	71.47%	69.04%	68.78%	68.34%	70.34%	70.24%
SVM	28.17%	26.52%	26.26%	24.34%	25.91%	26.86%	25.21%	27.73%	27.30%	28.60%	26.69%

## Data Availability

This study is an experimental analysis of a publicly available dataset. The data can be found in this web page: https://archive.ics.uci.edu/ml/datasets/Epileptic+Seizure+Recognition (accessed on 4 November 2022).

## References

[1] Amirmasoud A., Behroozi M., Shalchyan V., Daliri M.R. Classification of epileptic EEG signals by wavelet based CFC. Proceedings of the 2018 Electric Electronics, Computer Science, Biomedical Engineerings’ Meeting (EBBT).

[2] Patrick K., Brodie M.J. (2000). Early identification of refractory epilepsy. N. Engl. J. Med..

[3] Sylvia B., Garg L., Audu E.E. A novel method of EEG data acquisition, feature extraction and feature space creation for early detection of epileptic seizures. Proceedings of the 2016 38th Annual International Conference of the IEEE Engineering in Medicine and Biology Society (EMBC).

[4] Shoeibi A., Ghassemi N., Khodatars M., Jafari M. (2021). Applications of epileptic seizures detection in neuroimaging modalities using deep learning techniques: Methods, challenges, and future works. arXiv.

[5] Beeraka S.M., Kumar A., Sameer M., Ghosh S., Gupta B. (2021). Accuracy Enhancement of Epileptic Seizure Detection: A Deep Learning Approach with Hardware Realization of STFT. Circuits, Syst. Signal Process..

[6] Wang B., Guo J., Yan T., Ohno S., Kanazawa S., Huang Q., Wu J. (2016). Neural Responses to Central and Peripheral Objects in the Lateral Occipital Cortex. Front. Hum. Neurosci..

[7] Yan T., Dong X., Mu N., Liu T., Chen D., Deng L., Wang C., Zhao L. (2018). Positive Classification Advantage: Tracing the Time Course Based on Brain Oscillation. Front. Hum. Neurosci..

[8] Ren G.-P., Yan J.-Q., Yu Z.-X., Wang D., Li X.-N., Mei S.-S., Dai J.-D., Li Y.-L., Wang X.-F., Yang X.-F. (2017). Automated Detector of High Frequency Oscillations in Epilepsy Based on Maximum Distributed Peak Points. Int. J. Neural Syst..

[9] Sun C., Cui H., Zhou W., Nie W., Wang X., Yuan Q. (2019). Epileptic seizure detection with EEG textural features and imbalanced classification based on EasyEnsemble learning. Int. J. Neural Syst..

[10] Cogan D., Birjandtalab J., Nourani M., Harvey J., Nagaraddi V. (2016). Multi-Biosignal Analysis for Epileptic Seizure Monitoring. Int. J. Neural Syst..

[11] Zhang J., Zou J., Wang M., Chen L., Wang C., Wang G. (2013). Automatic detection of interictal epileptiform discharges based on time-series sequence merging method. Neurocomputing.

[12] Aarabi A., Fazel-Rezai R., Aghakhani Y. (2009). A fuzzy rule-based system for epileptic seizure detection in intracranial EEG. Clin. Neurophysiol..

[13] Boashash B., Azemi G. (2014). A review of time–frequency matched filter design with application to seizure detection in multichannel newborn EEG. Digit. Signal Process..

[14] Subasi A., Gursoy M.I. (2010). EEG signal classification using PCA, ICA, LDA and support vector machines. Expert Syst. Appl..

[15] Asif U., Roy S., Tang J., Harrer S. (2020). SeizureNet: Multi-spectral deep feature learning for seizure type classification. Machine Learning in Clinical Neuroimaging and Radiogenomics in Neuro-Oncology.

[16] Acharya U.R., Oh S.L., Hagiwara Y., Tan J.H., Adeli H. (2018). Deep convolutional neural network for the automated detection and diagnosis of seizure using EEG signals. Comput. Biol. Med..

[17] Antoniades A., Spyrou L., Took C.C., Sanei S. Deep learning for epileptic intracranial EEG data. Proceedings of the 2016 IEEE 26th International Workshop on Machine Learning for Signal Processing (MLSP).

[18] Hussein R., Palangi H., Ward R., Wang Z.J. (2018). Epileptic seizure detection: A deep learning approach. arXiv.

[19] Tsiouris K.M., Pezoulas V.C., Zervakis M., Konitsiotis S., Koutsouris D.D., Fotiadis D.I. (2018). A Long Short-Term Memory deep learning network for the prediction of epileptic seizures using EEG signals. Comput. Biol. Med..

[20] Ullah I., Hussain M., Qazi E.-U., Aboalsamh H. (2018). An automated system for epilepsy detection using EEG brain signals based on deep learning approach. Expert Syst. Appl..

[21] Lin Q., Ye S.-Q., Huang X.-M., Li S.-Y., Zhang M.-Z., Xue Y., Chen W.-S. (2016). Classification of epileptic EEG signals with stacked sparse autoencoder based on deep learning. Int. Conf. Intell. Comput..

[22] Zhang Q., Yang L.T., Chen Z., Li P. (2018). A survey on deep learning for big data. Inf. Fusion.

[23] Wei X., Zhou L., Chen Z., Zhang L., Zhou Y. (2018). Automatic seizure detection using three-dimensional CNN based on multi-channel EEG. BMC Med. Inform. Decis. Mak..

[24] Gers F.A., Schmidhuber J., Cummins F. (2000). Learning to Forget: Continual Prediction with LSTM. Neural Comput..

[25] Hochreiter S. (1998). The Vanishing Gradient Problem During Learning Recurrent Neural Nets and Problem Solutions. Int. J. Uncertain. Fuzziness Knowl. Based Syst..

[26] Andrzejak R.G., Lehnertz K., Mormann F., Rieke C., David P., Elger C.E. (2001). Indications of nonlinear deterministic and finite-dimensional structures in time series of brain electrical activity: Dependence on recording region and brain state. Phys. Rev. E.

[27] Howard A.G., Zhu M., Chen B., Kalenichenko D., Wang W., Weyand T., Andreetto M., Adam H. (2017). Mobilenets: Efficient convolutional neural networks for mobile vision applications. arXiv.

[28] Kingma D.P., Ba J. (2014). Adam: A method for stochastic optimization. arXiv.

[29] Michelucci U. (2018). Applied Deep Learning: A Case-Based Approach to Understanding Deep Neural Networks.

[30] Hartmann M., Koren J., Baumgartner C., Duun-Henriksen J., Gritsch G., Kluge T., Perko H., Fürbass F. (2022). Seizure detection with deep neural networks for review of two-channel electroencephalogram. Epilepsia.

[31] Abou Jaoude M., Jing J., Sun H., Jacobs C.S., Pellerin K.R., Westover M.B., Cash S.S., Lam A.D. (2020). Detection of mesial temporal lobe epileptiform discharges on intracranial electrodes using deep learning. Clin. Neurophysiol..

[32] de Jong J., Cutcutache L., Page M., Elmoufti S., Dilley C., Fröhlich H., Armstrong M. (2021). Towards realizing the vision of precision medicine: AI based prediction of clinical drug response. Brain.

[33] Gleichgerrcht E., Keller S., Drane D.L., Munsell B.C., Davis K.A., Kaestner E., Weber B., Krantz S., Vandergrift W.A., Edwards J.C. (2020). Temporal Lobe Epilepsy Surgical Outcomes Can Be Inferred Based on Structural Connectome Hubs: A Machine Learning Study. Ann. Neurol..

